# Hepatic fat and abdominal adiposity in early pregnancy together predict impaired glucose homeostasis in mid-pregnancy

**DOI:** 10.1038/nutd.2016.39

**Published:** 2016-09-19

**Authors:** L R De Souza, H Berger, R Retnakaran, P A Vlachou, J L Maguire, A B Nathens, P W Connelly, J G Ray

**Affiliations:** 1 Department of Obstetrics and Gynecology, St Michael's Hospital, Toronto, ON, Canada; 2 Institute of Medical Science, University of Toronto, Toronto, ON, Canada; 3 Keenan Research Centre for Biomedical Science of St Michael's Hospital, Toronto, Canada; 4 Lunenfeld-Tanenbaum Research Institute, Mount Sinai Hospital, Toronto, ON, Canada; 5 Department of Medical Imaging, St Michael's Hospital, Toronto, ON, Canada; 6 Department of Surgery, Sunnybrook Health Sciences Center, Toronto, ON, Canada; 7 Department of Health Policy Management Evaluation, University of Toronto, Toronto, ON, Canada; 8 Department of Medicine, St Michael's Hospital, Toronto, ON, Canada

## Abstract

Hepatic fat and abdominal adiposity individually reflect insulin resistance, but their combined effect on glucose homeostasis in mid-pregnancy is unknown. A cohort of 476 pregnant women prospectively underwent sonographic assessment of hepatic fat and visceral (VAT) and total (TAT) adipose tissue at 11–14 weeks' gestation. Logistic regression was used to assess the relation between the presence of maternal hepatic fat and/or the upper quartile (Q) of either VAT or TAT and the odds of developing the composite outcome of impaired fasting glucose (IFG), impaired glucose tolerance (IGT) or gestational diabetes mellitus at 24–28 weeks' gestation, based on a 75 g OGTT. Upon adjusting for maternal age, ethnicity, family history of DM and body mass index (BMI), the co-presence of hepatic fat and quartile 4 (Q4) of VAT (adjusted odds ratio (aOR) 6.5, 95% CI: 2.3–18.5) or hepatic fat and Q4 of TAT (aOR 7.8 95% CI 2.8–21.7) were each associated with the composite outcome, relative to women with neither sonographic feature. First-trimester sonographic evidence of maternal hepatic fat and abdominal adiposity may independently predict the development of impaired glucose homeostasis and GDM in mid-pregnancy.

## Introduction

Obesity in pregnancy has many consequences, including maternal insulin resistance (IR) and gestational diabetes mellitus (GDM).^1, 2^ Non-alcoholic fatty liver disease (NAFLD) and elevated abdominal adiposity are manifestations of the metabolic syndrome.^3^ Insulin resistance leads to a failure to suppress free fatty acids, which in turn, along with proinflammatory cytockines liberated from visceral adipose tissue (VAT), results in NAFLD.^4, 5^ We have previously shown that VAT and total adipose tissue (TAT) are each associated with IR in early pregnancy^6^ and impaired glucose homeostasis in mid-pregnancy,^7^ independent of BMI and other conventional risk factors for GDM.

While VAT and TAT are a reflection of IR, the co-presence of elevated abdominal AT and hepatic fat has not been evaluated in relation to dysglycemia and GDM at 24–28 weeks' gestation, the recommended time to screen for GDM. Accordingly, we tested the hypothesis that non-diabetic women with sonographic evidence of hepatic fat and elevated VAT or TAT in early pregnancy would be at highest risk of dysglycemia and GDM in mid-pregnancy.

## Research design and methods

This prospective cohort study was completed at a general obstetrics outpatient clinic at St Michael's Hospital in Toronto, Ontario, Canada. The study was approved by the Research Ethics Board of St Michael's Hospital, and participants provided written informed consent.

Healthy women aged 18 years and older were eligible for the study entry if they had a viable singleton pregnancy at 11–14 weeks' gestation. To reduce confounding effects, we excluded women with known pre-pregnancy DM (prior GDM), a prior history of polycystic ovarian syndrome, metformin use, ovulation induction or other infertility treatment, use of corticosteroids for autoimmune conditions or other medical co-morbidities on medications, or any chronic or pregnancy-specific disorder that might affect liver function, such as viral hepatitis.

Visceral abdomen tissue and TAT depth quartiles (Q) were each determined by ultrasound at 11–14 weeks' gestation (at the time of routine assessment of fetal nuchal translucency), using a reliable and validated protocol, as described elsewhere.^5, 6^ Sonographic assessment of hepatic fat (present or absent) was determined in a standardized fashion in the sagittal plane, which allows single image capture of the liver and adjacent right kidney. Two sonographers independently reviewed the hard copies of each ultrasound image and used a semi-quantitative scoring method to assess hepatic fat based on the presence of any of the following: (i) diffusely increased echogenic (‘bright') liver relatively greater than the right kidney (that is, ‘hepato-renal contrast'), and/or (ii) impaired visualization (blurring) of the portal and hepatic veins.^5^ This approach has a within-observer reliability of 0.95 and a between-observer reliability of 0.95, a sensitivity for NAFLD of 60–95%, and a specificity of 84–100%.^3^ Measurements were obtained using a Phillips IU22 and a GE E8 ultrasound machineswith either a 5–2 MHz or 9 MHz probe.^6^

At the time of the ultrasound measures, we collected information about maternal age, ethnicity (Caucasian, Black, South Asian, East Asian or Other), self-reported pre-gestational height and weight, and a history of type 2 DM among a first-degree relative. Weight at 11–14 weeks was directly measured using a calibrated scale.

A 75 g glucose tolerance test was performed at 24–28 weeks gestation, after an overnight fast. We defined impaired glucose homeostasis as a composite of impaired fasting glucose (IFG: fasting glucose⩾5.3 mmol l^−1^), impaired glucose tolerance (IGT: 1 h glucose⩾10.6 mmol l^−1^ or 2 h glucose⩾8.9 mmol l^−1^) or GDM (⩾ 2 abnormal glucose values, namely, fasting⩾5.3 mmol l^−1^, 1 h⩾10.6 mmol l^−1^ and/or 2 h⩾8.9 mmol l^−1^).^2^

### Data analysis

Multivariable logistic regression analysis was used to assess the association between the combination of maternal hepatic fat and/or Q4 of VAT and the composite outcome. Participants without sonographic evidence of hepatic fat and within Q1–Q3 of VAT served as the referent. The same model was created for the combination of hepatic fat and Q4 of TAT. Odds ratios were adjusted (aOR) for maternal age (continuous in years), ethnicity (Caucasian vs Non-Caucasian), any first-degree relative with type 2 DM, measured BMI at 11–14 weeks gestation (continuous in kg m^−2^) and change in BMI from 11–14 weeks to 24–28 weeks' gestation (continuous in kg m^−2^).

Statistical analyses were performed using SAS (version 9.1.3; SAS Institute, Cary, NC, USA).

## Results

There were 476 women analyzed, at a mean (s.d.) age of 32.9 (4.8) years (Table 1). BMI at 11–14 weeks gestation ranged from 17.2 to 49.9 kg m^−2^, with a mean (s.d.) of 25.1 (5.1) kg m^−2^. A total of 50 out of 476 women (10.5%) developed the composite of IFG, IGT or GDM.

The combined presence of hepatic fat and Q4 of VAT was associated with a higher risk of impaired glucose homeostasis (aOR 6.5, 95% CI: 2.3 to 18.5) (Table 2). In contrast, those women without hepatic fat on ultrasound but whose VAT was at Q4, had a marginally higher risk of the composite outcome (aOR 2.3, 95% CI: 1.0 to 5.4). For the combined presence of hepatic fat and Q4 of TAT on ultrasound, the aOR was 7.8 (95% CI 2.8 to 21.7).

## Conclusions

First-trimester maternal hepatic fat, in combination with a high VAT or TAT depth, predicted impaired glucose homeostasis at 24–28 weeks' gestation, independent of maternal age, ethnicity, family history of type 2 DM or maternal BMI.

Our study strength was the inclusion of a relatively large, multi-ethnic sample of women, followed prospectively from the first trimester of pregnancy. In addition, use of a standardized sonographic protocol, coinciding with prenatal measurement of fetal nuchal translucency, afforded a practical timepoint to assess AT and hepatic fat. As a limitation, ultrasonography was only able to identify hepatic fat in a qualitative manner, and could not detect small amounts of hepatic steatosis and the stages of NAFLD.^3, 8^

VAT appeared to be an independent predictor of type 2 DM and the metabolic syndrome.^9, 10^ Excess VAT involves greater release of free fatty acids into the portal circulation along with proinflammatory cytokines,^9, 10^ and manifests as hepatic fat and inflammation that defines the spectrum of NAFLD.^9^ NAFLD has been shown to be more prevalent in non-pregnant women with previous GDM (38%, 95% CI: 28 to 47) than those without previous GDM (17%, 95% CI: 10 to 24).^11^ Using linked administrative datasets in Sweden, women with a coded diagnosis of NAFLD before pregnancy had a higher risk of GDM than those without a coded diagnosis of NAFLD (adjusted relative risk 2.78, 95% CI: 1.25–6.15).^12^ Thus, preliminary evidence suggests that women with excess hepatic fat may be at higher risk of GDM.

Research on objectively measured central obesity and impaired glucose homeostasis in pregnancy is also limited. We previously found that VAT and TAT respectively contributed to 23 and 25% of the variance in IR during the first trimester of pregnancy.^6^ More recently, we showed that Q4 of TAT depth, and especially Q4 of VAT depth, were associated with dysglycemia and GDM.^7^ What was not known is whether there is an additive effect when high VAT or TAT depth are considered in conjunction with the presence of hepatic fat. The current study findings suggest that there is an additive effect, and the reason may be that individuals with high VAT or TAT in conjunction with hepatic fat may have a pathological predisposition to IR. Among the 355 women in the VAT Q1–3 group, 48 (13.5%) had identified hepatic fat, whereas, among the 28 women in the VAT Q4 group, only 11 (39.3%) had sonographic signs of hepatic fat, resulting in a six times higher odds.

Assessing for hepatic fat in combination with VAT or TAT, using first-trimester ultrasonography, may offer a promising method of early detection of IR and a woman's predisposition to GDM. The sonographic measurement of hepatic fat and adipose tissue in early pregnancy could be a cost-effective method easily added to routine, early antenatal screening. Women identified as having elevated hepatic fat and VAT or TAT during routine antenatal care at 11–14 weeks' gestation, could then be identified in early pregnancy as having a predisposition to IR, and accordingly, might be offered early glucose testing, in addition to efficacious interventions that reduce their risk of GDM and related perinatal complications — something that must be proven within large scale prospective studies.^13^

## Figures and Tables

**Table 1:** Characteristics of the 476 study participants and the study measures

*Measure*	*Value*
*At 11–14 weeks gestation*	
Mean (s.d.) age at time of enrolment, years	32.9 (4.8)
No. (%) with a first degree relative with type 2 diabetes mellitus	110 (22.7)
No. (%) Caucasian	251 (51.8)
Mean (s.d.) BMI, kg m^−2^	25.1 (5.1)
No. (%) with parity⩾1	208 (43.7)
	
*At 24–28 weeks gestation*	
Mean (s.d.) net change in BMI from 11-14 to 24–28 weeks, kg m^−2^	2.6 (1.8)
No. (%) meeting the criteria for IFG, IGT or GDM^a^	50 (10.5)
No. (%) meeting the criteria for GDM^b^	43 (9.0)

Abbreviations: BMI, body mass index; OGTT, oral glucose tolerance test; GDM, gestational diabetes mellitus; IFG, impaired fasting glucose; IGT, gestational impaired glucose tolerance. IGT was based on an abnormal glucose value at 1 h⩾10.6 mmol l^−1^ or 2 h⩾8.9 mmol l^−1^, in isolation.

a IFG was based on an abnormal fasting value⩾5.3 mmol l^−1^, in isolation.

b GDM was based on the presence of at least two abnormal serum glucose values: fasting⩾5.3 mmol l^−1^; 1 h⩾10.6 mmol l^−1^ and/or 2 h⩾8.9 mmol l^−1^

**Table 2:** Development of the composite outcome of impaired fasting glucose (IFG), impaired glucose tolerance (IGT) or GDM, in relation to the presence or absence of maternal hepatic fat in combination with lower quartiles (Q1-3)^a^ vs the highest quartile (Q4)^a^ of visceral adipose tissue depth (VAT) or total adipose tissue depth (TAT), at 11-14 weeks gestation

*Exposure*	*Number (%) with outcome*	*Adjusted odds ratio (95% confidence interval)*^b^
Hepatic fat absent, VAT Q1-3 (*n=*307)	18 (5.9)	1.00 (referent)
Hepatic fat present, VAT Q1-3 (*n=*48)	4 (8.3)	1.4 (0.45 to 4.5)
Hepatic fat absent, VAT Q4 (*n=*92)	17 (18.5)	2.3 (1.0 to 5.4)
Hepatic fat present, VAT Q4 (*n=*29)	11 (37.9)	6.5 (2.3 to 18.5)
Hepatic fat absent, TAT Q1-3 (*n=*307)	18 (5.9)	1.00 (referent)
Hepatic fat present, TAT Q1-3 (*n=*49)	3 (6.1)	0.98 (0.27 to 3.5)
Hepatic fat absent, TAT Q4 (*n=*92)	17 (18.5)	2.2 (0.92 to 5.2)
Hepatic fat present, TAT Q4 (*n=*28)	12 (42.9)	7.8 (2.8 to 21.7)

Abbreviations: IFG, mpaired fasting glucose; IGT, impaired glucose tolerance.

a VAT Q1–3:⩽4.8 cm; VAT Q4:>4.8 cm. TAT Q1–3:⩽7.0 cm; TAT Q4:>7.0 cm.

b Adjusted for maternal age at delivery, ethnicity, family history of type 2 DM among first degree relatives, BMI at 11–14 weeks' gestation, change in BMI from 11–14 weeks' to 24–28 weeks' gestation.
